# Yeast species isolated from Texas High Plains vineyards and dynamics during spontaneous fermentations of Tempranillo grapes

**DOI:** 10.1371/journal.pone.0216246

**Published:** 2019-05-02

**Authors:** Matthias Bougreau, Kenia Ascencio, Marie Bugarel, Kendra Nightingale, Guy Loneragan

**Affiliations:** 1 Department of Plant and Soil Sciences, Texas Tech University, Lubbock, Texas, United States of America; 2 Department of Animal and Food Sciences, International Center for Food Industry Excellence, Texas Tech University, Lubbock, Texas, United States of America; University of Torino, ITALY

## Abstract

Vineyards and grape musts harbor complex locally specific microbial communities, among which yeast species can be responsible of spontaneous alcoholic fermentation. Although relying on indigenous yeast can be a risk for winemaking, local yeast diversity is associated with complexity and stronger identity of the wine produced, compared to inoculated alcoholic fermentation with commercial yeast strains. In this context, the main yeast species present on grapes, leaves and soils of Tempranillo and Cabernet Sauvignon vineyards in the hot semi-arid climate of the Texas High Plains area were investigated, as well as the presence and dynamics of yeast species during spontaneous fermentations of Tempranillo grapes from the same vineyards. Molecular characterization of yeast species was performed using culture-dependent 5.8S-ITS restriction fragment length polymorphism method and sequencing. Yeast species recovered from grapes, leaves, and soils were mainly dominated by *Aureobasidium pullulans*, *Cryptococcus* species, *Filobasidium* species and *Naganishia* species, typical members of the vineyard environment. One isolate of potential enological interest, *Lachancea thermotolerans*, a fermenting yeast with potential in must acidification, was recovered from the vineyard environment. However, spontaneous alcoholic fermentations revealed the presence of fermenting yeast species, including *Saccharomyces cerevisiae*, *Lachancea thermotolerans* and *Hanseniaspora* species. The presence of the three aforementioned species is of extreme interest for winemaking in the Texas High Plains area. Indeed, *Saccharomyces cerevisiae* is the model for alcoholic fermentation, *Hanseniaspora* species have been shown to improve palatability of wines, and *Lachancea thermotolerans* has become of increasing interest due to its potential to acidify musts and palatability. One of the main characteristics of grapes grown in the Texas High Plains area being the lack of acidity, focusing on these three yeast species could promote the development of locally oriented started cultures for the production of wines with a stronger local identity.

## Introduction

Wine is an alcoholic beverage made from fermented grape juice, and consequently requires microorganisms to convert grape sugars into alcohol and carbon dioxide. Winemakers perform inoculated fermentations by adding commercial active dry yeast (ADY) preparations to the must, or alternatively rely on the indigenous yeast populations present in the vineyard or the winery environment to perform spontaneous fermentations [1]. The yeast *Saccharomyces cerevisiae* is the model for alcoholic fermentation, and most of the ADY strains commercially available are *S*. *cerevisiae*. *S*. *cerevisiae* strains offer good control of the fermentation kinetics and desired aromatic impact to the wine [2, 3]. However, inoculated fermentation of grape juice with *S*. *cerevisiae* strains can result in a decreased wine complexity in comparison to spontaneous fermentations [4, 5], and has been associated with a decreased environmental biodiversity [6]. It is also recognized that the use of the same commercial strains in wineries all over the world could decrease the diversity of wines and lead to a globalization of wine sensory properties, due to the specific aromatic compounds released during fermentation by yeast strains [7].

Spontaneous fermentations on the other hand offer more diversity due to the many yeast species present in the vineyard and winery environments. Non-*Saccharomyces* yeasts such as *Candida*, *Debaryomyces*, *Dekkera*, *Hanseniaspora*, *Hansenula*, *Issatchenkia*, *Lachancea*, *Metschnikowia*, *Pichia*, *Rhodotorula*, *Schizosaccharomyces*, *Torulaspora* and *Zygosaccharomyces* have been found on grape berries or in the environment of wineries [8–11]. Although they can be considered spoilage microorganisms, species such as *Hanseniaspora uvarum*, *Lachancea thermotolerans*, *Metschnikowia pulcherrima*, *Schizosaccharomyces pombe* and *Torulaspora delbrueckii* have also been shown to improve sensory properties of wines, especially when used in combination with *S*. *cerevisiae* or other *Saccharomyces* species, when compared to inoculation of *S*. *cerevisiae* alone [12–21]. Although not involved in the completion of the alcoholic fermentation process, non-*Saccharomyces* strains can produce positive volatile metabolites during the early stages of alcoholic fermentation [5]. At later stages of the alcoholic fermentation, *S*. *cerevisiae* dominates the non-*Saccharomyces* strains and complete the alcoholic fermentation [22].

The presence of enological yeast species in the vineyard environment, and especially on the surface of the berries, has been suggested to be influenced by the climate, grape variety, and specific viticulture practices, as well as to correlate with the chemical composition of wine and thus regional wine identity [11, 23–25]. Winemakers would benefit from increased knowledge of the indigenous microbial populations inhabiting their local grape environment. Using indigenous yeast for fermentations could preserve the microbial component of local terroir specificities, and thus allow for a stronger local wine identity, instead of relying solely on commercial ADY strains isolated from vineyards with different geographical features.

The aim of this study was to investigate the presence of indigenous yeasts in some vineyards of the Texas High Plains American Vitivulture Area (AVA), a new and fast-growing area for viticulture. In this hot semi-arid climate, we selected for study the two most planted *Vitis vinifera* red grape varieties, Tempranillo and Cabernet Sauvignon. This study was conducted in two separate vintages in 2014 and 2015, with two different approaches. During the 2014 vintage, we focused our research on the evaluation of the diversity of the yeast populations present on grapes, leaves and soil in two different vineyards both growing Cabernet Sauvignon and Tempranillo grapes. During the 2015 vintage, we focused on spontaneous fermentations of Tempranillo grapes from the same vineyards studied in 2014 to investigate the presence and dynamics of indigenous yeast species during alcoholic fermentations.

## Materials and methods

### Vineyard locations for sampling

Two distinct vineyards in the Texas High Plains were sampled: vineyard A (Newsom Vineyards, Plains, Yoakum County, TX, USA, coordinates 33.272579, -102.787675) and vineyard B (Reddy Vineyards, Brownfield, Terry County, TX, USA, coordinates 33.184424, -102.236613). The two vineyards are located approximately 35 miles away from each other. Within each vineyard, adjacent parcels of Cabernet Sauvignon and Tempranillo were sampled. The same vineyards were sampled during the two phases of the study, in 2014 and 2015. Sampling in both vineyards was allowed by the respective owners Neal Newsom and Vijay Reddy.

### Sampling and isolation for vineyard environmental yeasts analysis

For evaluation of the biodiversity of yeast in the vineyard environment, samples were taken one day prior to harvest during the 2014 vintage. Each parcel was divided into ten equal length sections and one vine was randomly selected from each section. Berries, leaves and proximal soil samples were collected for each selected vine. Soil samples were taken with sterile single-use spoons and directly placed into sealed sterile bags. Five to ten leaves were collected manually with sterile gloves and placed directly into sealed sterile bags. Ten to twenty sound berries were randomly collected manually with sterile gloves and placed directly into sealed sterile bags. Samplers changed gloves between each sampling to avoid potential cross contamination. As a result, 30 samples were taken for each of the four parcels, and a total of 120 samples were collected during this study. All samples were placed directly into a cooler with ice packs for transportation to the laboratory and microbiological analysis. Samples were processed directly upon arrival to the laboratory. Approximately 20 grams of soil, 2 grams of leaves, and between 5 to 10 grams of berries (five berries) were weighed for each sample and placed individually into 50 ml conical tubes, followed by the addition of 25 ml of 0.1% peptone water. Each tube was vortexed at high speed for 1 minute and 1:10 serial dilutions were directly prepared in 0.1% peptone water. Aliquots of sample homogenate (0.1 ml) were plated in triplicate on Yeast Extract Peptone Dextrose (YPD, Difco, Fisher Bioblock Scientific, Illkirch, France) agar plates supplemented with 50 mg/L chloramphenicol (Sigma-Aldrich, St. Louis, MO, USA), and incubated at 30°C for two to five days. Colonies were enumerated and up to 50 colonies per sample were randomly picked and sub-cultured on YPD agar plates and incubated at 30°C for three to five days. Isolated colonies were then transferred to and grown in YPD broth at 30°C for two to five days then stored at -80°C after addition of 15% glycerol (Ibi Scientific, Peosta, IA, USA).

### Sampling and isolation for analysis of yeast evolution during spontaneous fermentations

Spontaneous fermentations were conducted using grapes collected during the 2015 vintage. The grapes were collected one day prior to commercial harvest. Due to logistic and weather issues, Cabernet Sauvignon grapes were not available for sampling, and only Tempranillo grapes were sampled for this part of the study. The Tempranillo vines harvested belonged to the same vineyard sections defined for the 2014 sampling. Whole clusters were collected using sterile gloves and placed directly into single closed sterile bags. Each cluster was then weighed and placed into a cooler with ice packs for transportation to the laboratory. A total of 19.3 kg of Tempranillo grapes were collected from vineyard A (108 clusters, average cluster weight 0.178 g), and 21.3 kg of Tempranillo grapes were collected from vineyard B (72 clusters, average cluster weight 0.296 g). Fermentation vats were coded batch A and batch B.

Upon arrival to the laboratory, clusters were de-stemmed manually in sterile conditions and crushed into sanitized stainless-steel tanks (20 gallons Wineasy fermenters, Blichmann Engineering, Lafayette, IN, USA) with a sanitized stainless steel smasher (one per batch to avoid cross contamination). After crushing, the must was stirred and 40 ml was transferred into a 50 ml conical tube and frozen at -80°C for microbial analysis. Brix content by refractometry and pH were measured after homogenization of each batch. Batch A presented a 26.1 Brix and a pH of 3.89, while batch B presented a 23.2 Brix and a pH of 4.03. Daily and prior to any sampling, musts were punched down and homogenized using the pre-sanitized stainless steel smashers. Spontaneous fermentations were monitored by measuring temperature and alcohol content by ebulliometry daily. Final glucose/fructose concentrations at the end of fermentations were measured by enzymatic method (product no. 4A145, Vintessential Laboratories, Dromana, VIC, Australia). Temperature was room controlled and temperatures in fermenters were kept between 19°C and 23°C at the peak of fermentation. At day 0 (day of harvest) and until end of fermentations, aliquots of 40 ml were regularly sampled from each batch and transferred in 50 ml conical tubes and processed for microbiological analysis. Each tube was vortexed at high speed for 1 minute and 1:10 serial dilutions were directly prepared in 0.1% peptone water. Aliquots of sample homogenate (0.1 ml) were plated in triplicate on Yeast Extract Peptone Dextrose (YPD, Difco, Fisher Bioblock Scientific, Illkirch, France) agar plates supplemented with 50 mg/L chloramphenicol (Sigma-Aldrich, St. Louis, MO, USA), and incubated at 30°C for two to five days. Colonies were enumerated and 50 colonies were randomly picked and sub-cultured on YPD agar plates and incubated at 30°C for two to five days. Isolated colonies were then transferred to and grown in YPD broth at 30°C for two to five days then stored at -80°C after addition of 15% glycerol (Ibi Scientific, Peosta, IA, USA).

### Sanitation of fermentation equipment

Prior to use for fermentations, the stainless steel fermenters and the stainless steel smashers were cleaned and sanitized according to the following protocol. All containers and equipment to come in contact with must were thoroughly rinsed with pressurized water to remove any visible debris. A solution of 5g/L PBW (Five Star Chemicals, Commerce City, CO, USA) was prepared in water and poured into the fermenters to bath and clean all parts and smashers. Outside of fermenters were cleaned with the same solution. All items were then rinsed with hot water. A solution of 7g/L citric acid (L.D. Carlson, Kent, OH, USA) and 15g/L Potassium Metabisulfite (L.D. Carlson, Kent, OH, USA) was prepared with warm water and poured into the fermenters to bath and sanitize all parts and smashers. Outside of fermenters were sanitized with the same solution. All items were then rinsed thoroughly with water. The stainless steel smashers were cleaned and sanitized according to the same protocol daily prior and after use for homogenization and punching down.

### Yeast identification

Frozen cultures of yeast isolates recovered during the 2014 and 2015 vintages were streaked for growth on YPD agar plates, and incubated two to five days at 30°C, for confirmation of pure culture and selection of isolated colony. A single isolated colony was then used to inoculate 5 mL of YPD medium, and incubated overnight at 30°C. One ml of overnight culture was then used to perform DNA extractions using the InstaGene Matrix kit (Bio-Rad, Marnes-la-Coquette, France) following manufacturer recommendations. The diversity of the rDNA located between the 18S and 28S rRNA, including the internal transcribed spacers (ITS) ITS1 and ITS2, and the 5.8S subunit of the rRNA, was investigated as previously described [26, 27]. Briefly, the ITS fragments were amplified using primers ITS1 (5’-TCCGTAGGTGAACCTGCGG-3’) and ITS4 (5’-TCCTCCGCTTATTGATATGC-3’) at the final concentration of 0.5 μM and the GoTaq Master Mix (Promega, Madison, WI, USA) with the following thermal program: 5 min at 94°C, 40 cycles of 94° for 1 min, 55.5°C for 2 min, and 72°C for 2 min, and a final elongation at 72°C for 5 min. Amplification and size of amplicons were evaluated by migration/separation in 1.3% agarose gels. Amplicons were then purified using the GenElute PCR Clean Up kit (Sigma-Aldrich, St. Louis, MO, USA). Purified amplicons were quantified based on the absorbance of DNA at 260 nm with a NanoDrop 2000 (Thermofisher, Waltham, MA, USA). A total of 1 ng of purified amplicon was then used in two separate endonuclease restriction reactions using the *HaeIII* and *HinfI* enzymes (New England Biolabs, Ipswich, MA, USA), following manufacturer recommendations. The digestion products were then migrated on 3% agarose gels. Digestion profiles were interpreted and analyzed individually using BioNumerics v.6.6 software (Applied Maths, Belgium). Profile comparisons were performed for each enzyme individually using the following settings: Dice Band-based identification, 0.5% optimization, 0.5% tolerance, Unweighted Pair Group Method with Arithmetic Mean (UPGMA) cluster analysis, and with the cophenetic correlation for branch quality. Profiles combining the individual HaeIII and HinfI restriction patterns were determined, and the ITS1-ITS4 fragment of a single isolate belonging to each profile was selected as a profile-representative and sequenced. Sequences were compared *in silico* using the nucleotide Basic Local Alignment Search Tool (BLAST) algorithm (http://blast.ncbi.nlm.nih.gov/Blast.cgi) against the entire whole-genome shotgun contigs (wgs) database, in order to attribute a species identification to each profile.

### Diversity analysis

Richness was evaluated as the number of different species isolated from each sample type. Shannon’s index (H’) measures the degree of uncertainty to predict to what species an isolate chosen at random from a population will belong, and is calculated according to the following equation:

$$H^{\prime }=-\sum_{i=1}^{S}\left(p_{i}\;\text{ln}\;p_{i}\right)$$

where H’ is the Shannon diversity index, *p*_*i*_ the fraction of the entire population made up of species *i*, *S* the number of different species identified, and *Σ* the sum from species 1 to *S*. According to this formula, the higher the Shannon’s index is, the higher is the diversity of the sample type.

### Statistical analysis

A principal component analysis was performed based on the percentages of each yeast population recovered in an effort to visualize the grouping of samples by vineyard, variety and nature (soils, leaves and grapes). The principal component analysis was performed using freely available statistical analysis software R version 3.2.2 (R Core development team, 2015) with packages FactoMineR version 1.39 [28] and Factoextra version 1.0.5 [29].

## Results

### Yeast biodiversity on grapes, leaves and soil samples

Leaf samples showed on average the highest counts, ranging between 10^4^ and 10^6^ CFU/g, while soil samples ranged from 10^3^ to 10^5^ CFU/g (Table 1). Overall, grape samples showed the lowest counts, ranging between 10^2^ and 10^4^ CFU/g, with the exception of Tempranillo grapes sampled from vineyard A reaching 2.3x10^5^ CFU/g. The richness, measured as the number of different species recovered per sample type, was highest in soil samples, varying from 8 to 14, lowest in grape samples, varying from 2 to 7, and was not correlated with the number of isolates recovered, with a correlation coefficient of 0.09. The diversity was higher in soil samples and lower in grape samples, with a Shanon’s index between 1.73 and 2.17 for soil samples and between 0.22 and 1.07 for grape samples (Table 1). No difference in richness and diversity were attributable to the *Vitis vinifera* varieties sampled or the vineyards enrolled in this study.

**Table 1:** Yeast count and diversity indexes.

	ASC^a^	AST	BSC	BST	ALC	ALT	BLC	BLT	AGC	AGT	BGC	BGT
Total yeast counts (x10^4^CFU/g ± CI95%^b^)	0.82 ±0.21	8.14 ±4.27	0.98 ±0.45	1.08 ±0.36	12.58 ±5.85	24.69 ±17.1	6.28 ±2.54	4.57 ±2.12	0.24 ±0.08	22.81 ±4.55	0.34 ±0.15	0.06 ±0.01
Number of isolates	31	56	47	27	53	75	58	121	70	109	94	33
Richness^c^	11	10	14	8	7	8	10	6	3	7	3	2
Shanon’s Index^d^ (Species diversity)	2.09	1.80	2.17	1.73	1.47	1.20	1.41	0.53	0.46	1.07	0.51	0.22

^a^Three letter identification: first letter, vineyard (A or B); second letter, Nature(S, soils; L, leaves; G, grape berries); third letter, variety (C, Cabernet Sauvignon; T, Tempranillo).

^b^CI95%, 95% confidence interval.

^c^Richness corresponds to the number of different species isolated from each sample type.

^d^The higher the Shanon’s index is, the higher the diversity of the sample type is.

**Fig 1:**
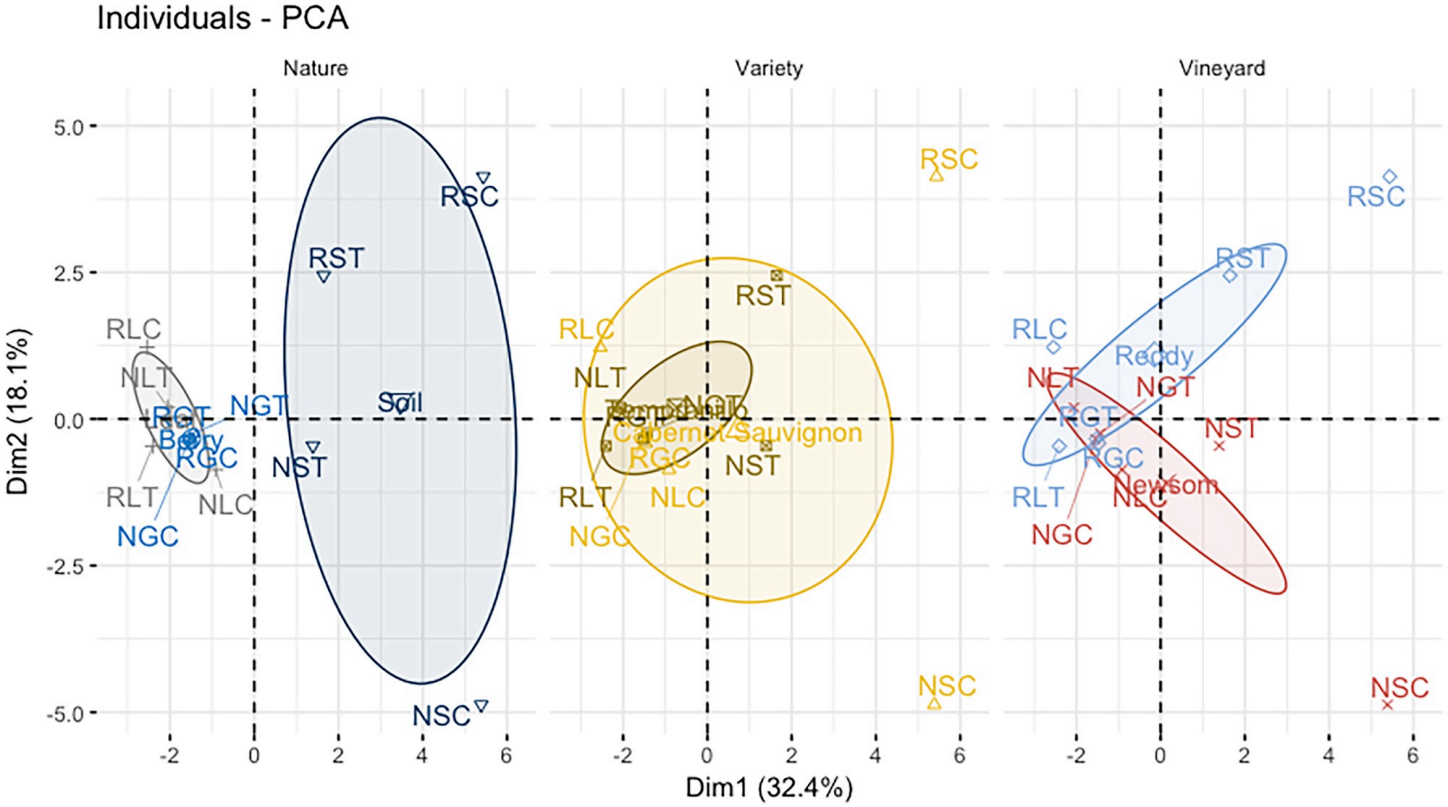
Principal component analysis of yeast diversity on 2014 samples. The spatial representation of sample types from the 2014 sampling of soils, leaves and grape berries according to the two first principal components (Dim1 and Dim2) built on the differences in yeast populations is presented three times. Nature (left) presents ellipses according to the nature of the sample: soils in red, leaves in gray and berries in blue. Variety (center) presents ellipses according to the variety of the parcel sampled: Cabernet Sauvignon in yellow and Tempranillo in light blue. Vineyard (right) presents ellipses according to the vineyard sampled: Vineyard A in dark blue and Vineyard B in brown. The first principal component (Dim 1, X axis) explains 30.0% of the total variance of the dataset, while the second principal component (Dim 2, Y axis) explains 17.7% of the total variance of the dataset.

Among the 799 isolates analyzed, 20 species belonging to 15 genera were identified (Table 2). The majority of the isolates identified on all types of samples belonged to three genera: *Aureobasidium* (483 isolates) represented by *A*. *pullulans* only; *Cryptococcus* (161 isolates) represented by *C*. *magnus*, *C*. *laurentii* and *Cryptococcus sp*.; and *Naganishia* (57 isolates) represented by *N*. *globosa*, *N*. *albida*, *N*. *friedmannii* and *Naganishia sp*. *A*. *pullulans* was predominantly identified on the leaf and berry samples. Species from the three genera *Solicoccozyma*, *Filobasidium* and *Papiliotrema* were identified only on two types of samples. *Solicoccozyma aeria* was predominantly recovered from soil (44 isolates), with only one isolate recovered from the berry samples. *Filobasidium* species (*F*. *floriforme* and *Filobasidium sp*.) were recovered from leaf and berry samples (19 isolates), and never from soil samples. *Papiliotrema terrestris* was mainly recovered from soil samples (7 isolates) with only one isolate identified from a leaf sample. The other species identified were recovered from only one type of sample (soil, leaf or berry). *Lecythophora* species (*Lecythophora canina*, 1 isolate, and *Lecythophora sp*., 4 isolates), *Rhodotorula* species (*R*. *nothofagi*, 2 isolates, *R*. *babjevae*, 1 isolate and *R*. *sp*., 2 isolates) and *Cystobasidium* species (*Cystobasidium minutum*, 3 isolates and *Cystobasidium sp*., 1 isolate), *Saitozoma flava* and *Lachancea thermotolerans* (1 isolate each) were only recovered from soil samples. *Sporisorium* species (*Sporisorium*. *fraserianum*, 3 isolates and *Sporisorium consanguineum*, 1 isolate), *Kwoniella dendrophila* (2 isolates), *Sporobolomyces beijingensis* (2 isolates) and *Vishniacozyma carnescens* (1 isolate) were only recovered from leaf samples. Principal component analysis (PCA) on yeast species per sample types allowed the discrimination of soil samples from leaf and grape berry samples on the first principal component (Fig 1). Moreover, the second principal component identified a vineyard dependent differentiation of soil samples. Berry and leaf samples were closely grouped by PCA and not differentiable from each other. The two *Vitis vinifera* grape varieties were not differentiated by PCA.

**Table 2:** Yeast species composition on grapes, leaves, and in soils, in percentage of isolates.

	ASC^a^	AST	BSC	BST	ALC	ALT	BLC	BLT	AGC	AGT	BGC	BGT
*Aureobasidium pullulans*	20.6	38.5	25.0	23.3	34.0	56.0	50.8	87.8	85.7	56.4	83.9	94.3
*Cryptococcus magnus*	5.9	6.2	5.8		37.7	26.7	28.8	8.1	12.9	32.7	14.0	5.7
*Cryptococcus laurentii*		4.6										
*Cryptococcus* sp.	8.8	1.5	5.8	20.0	1.9	1.3	1.7			5.5		
*Filobasidium floriforme*						10.7	6.8					
*Filobasidium* sp.					3.8		1.7		1.4	2.7		
*Naganishia globosa*	26.5	16.9	7.7	3.3	13.2	1.3				0.9	2.2	
*Naganishia albida*		4.6	7.7	6.7		1.3	3.4			0.9		
*Naganishia friedmannii*					1.9							
*Naganishia* sp.		6.2	1.9	3.3		1.3						
*Solicoccozyma aeria*	14.7	18.5	25	33.3	7.5					0.9		
*Papiliotrema terrestris*	2.9		7.7	6.7			1.7					
*Lecythophora canina*			1.9									
*Lecythophora* sp.	5.9	1.5	1.9									
*Rhodotorula nothofagi*	2.9		1.9									
*Rhodotorula babjevae*	2.9											
*Rhodotorula* sp.	5.9											
*Cystobasidium minuta*	2.9	1.5	1.9									
*Cystobasidium* sp.				3.3								
*Sporisorium fraserianum*								2.4				
*Sporisorium consanguineum*								0.8				
*Kwoniella dendrophila*							1.7	0.8				
*Sporobolomyces beijingensis*						1.3	1.7					
*Saitozoma flava*			1.9									
*Vishniacozyma carnescens*							1.7					
*Lachancea thermotolerans*			1.9									

^a^Three letter identification: first letter, vineyard (A or B); second letter, Nature(S, soils; L, leaves; G, grape berries); third letter, variety (C, Cabernet Sauvignon; T, Tempranillo).

### Main yeasts in must and evolution during spontaneous fermentations

**Fig 2:**
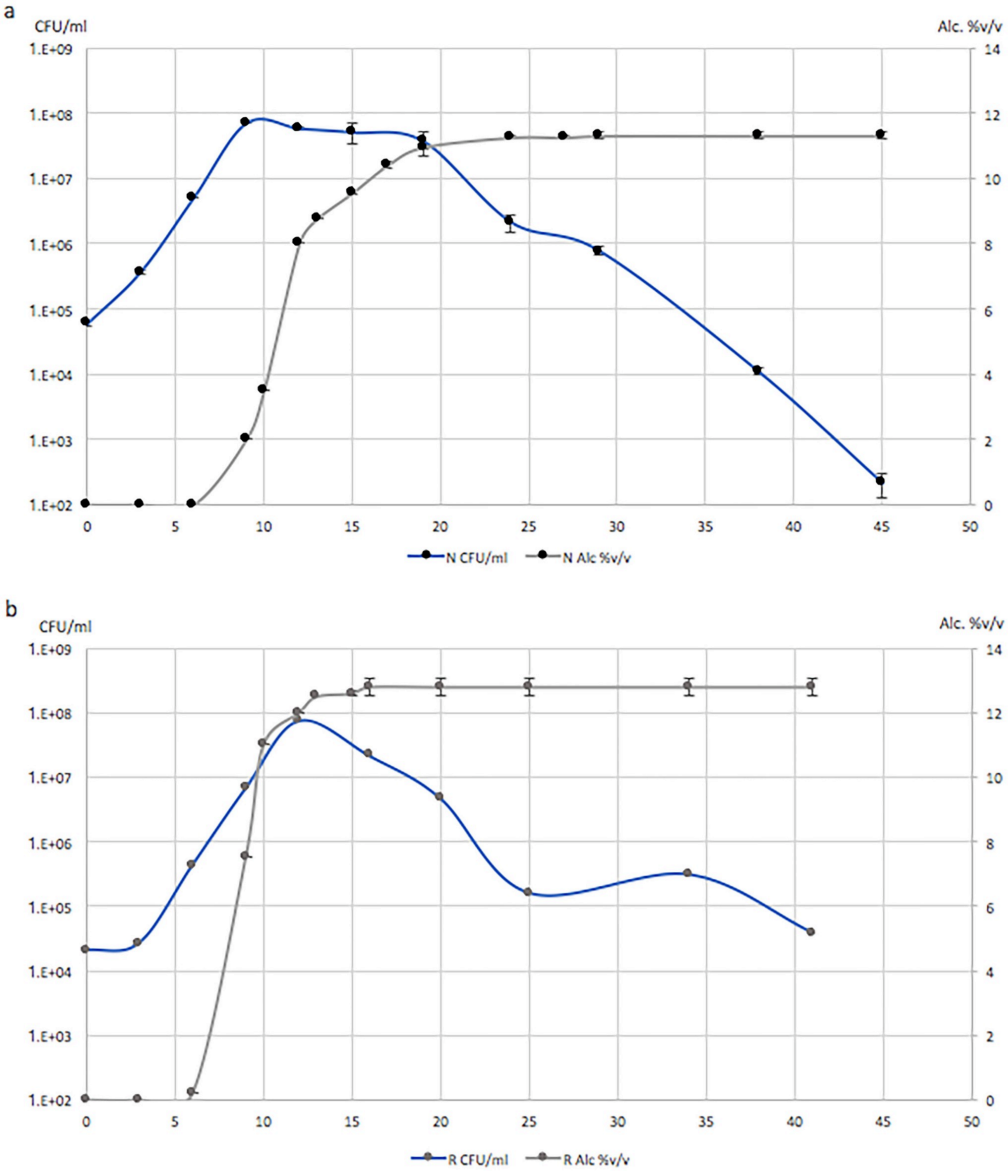
Monitoring and alcohol content during fermentations. Yeast concentration and alcohol content were evaluated by regular sampling of the must from batches A and B. Isolation of yeast was conducted by plating serial dilutions of the must on Yeast Peptone Dextrose agar medium. Triplicate dilution plates with 20 to 200 colonies were counted to estimate the yeast concentration in CFU/ml of must. Alcohol content was measured using a Malligand ebulliometer to display direct alcohol concentration. Fig 2a displays the yeast concentration (blue line) in CFU/ml (left Y axis) and the alcohol content (black line) in %v/v (right Y axis) after harvest and during fermentation of batch A. Fig 2b displays the yeast concentration (blue line) in CFU/ml (left Y axis) and the alcohol content (black line) in %v/v (right Y axis) after harvest and during fermentation of batch B. Error bars represent SEM.

During the 2015 vintage, clusters from the same Tempranillo vineyards A and B studied during the 2014 vintage were sampled. Upon arrival at the laboratory, these clusters were gently crushed into two separate sterile stainless-steel tanks for fermentations. Brix content and pH were measured after homogenization of each batch. In bactch A, the grapes from vineyard A were harvested at 26.1 Brix and a pH of 3.89, while in batch B the grapes from vineyard B were harvested at 23.2 Brix and a pH of 4.03. Fermentation occurred in both batches. The changes in yeast counts and alcohol content over time were monitored (Fig 2). In batch A, fermentation started between day six and day nine, and reached a final alcohol content of 11.6%v/v on day 29. There was a peak in yeast concentration observed at day 9, reaching 1x10^8^CFU/ml. The final glucose/fructose concentration, measured at day 45, was 36.77g/L. In batch B, the fermentation started between day three and day six, and reached a final alcohol content of 12.9%v/v on day 16. A peak in yeast concentration for the fermentation of vineyard B grapes was observed at day 12, similarly reaching approximately 1x10^8^CFU/ml with a final glucose/fructose concentration of 1.83g/L measured on day 42.

**Fig 3:**
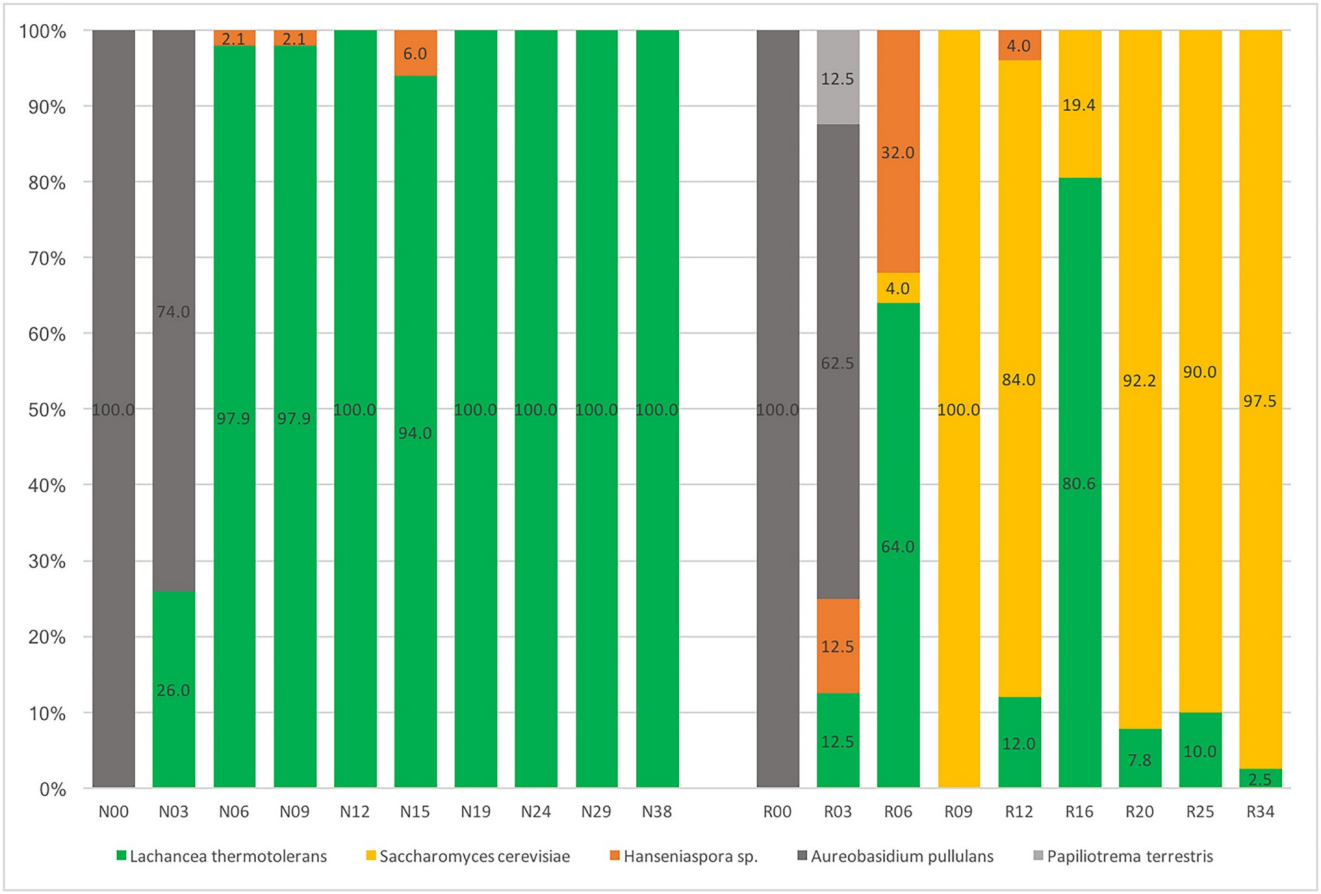
Dynamics of indigenous yeast species at harvest and along alcoholic fermentations. Percentages of each species recovered during monitoring of fermentations are presented for batches A (left) and B (right) for each sampling day at harvest (Day 0) and during fermentation. *Aureobasidium pullulans*, dark gray; *Papillotrema terrestris*, light gray; *Lachancea thermotolerans*, green; *Hanseniaspora species*, orange; *Saccharomyces cerevisiae*, yellow.

Among the 648 isolates analyzed at harvest and along alcoholic fermentations, four different species were recovered from the two batches: *Aureobasidium pullulans*, *Hanseniaspora sp*., *Lachancea thermotolerans* and *Saccharomyces cerevisiae* (Fig 3). One isolate of a fifth species, *Papiliotrema terrestris*, was also recovered from batch B on day three. The yeast population was dominated in both batches by *A*. *pullulans* at days zero and three, with respectively 100% and 74% of the isolates recovered from batch A, and 100% and 62.5% from batch B. After day three, corresponding to the start of alcoholic fermentation, *A*. *pullulans* was never re-isolated. *Hanseniaspora sp*. were always recovered as a minor fraction of the yeast population, on day six, nine and 15 from batch A (respectively 2.1%, 2.1% and 12.0% of the isolates) and on days three, six and 12 from batch B (respectively 12.5%, 32.0% and 4%). In batch A, *L*. *thermotolerans* was first recovered on day three (13% of the isolates) then became the dominant yeast species recovered from all samples after the start of fermentation, representing 97.9% of the isolates at day six and nine, 100% at day 12, 94.0% at day 15, and was the only species isolated after day 15. In batch B, *L*. *thermotolerans* was also recovered first on day three (12.5% of the isolates), then became the dominant yeast species on day six (64.0%). *S*. *cerevisiae*, first recovered on day 6 (4% of the isolates), became then the dominant species, representing all the isolates recovered on day nine and 84% on day 12. After day 12, only *L*. *thermotolerans* and *S*. *cerevisiae* were isolated from the samples. *L*. *thermotolerans* became dominant again on day 16 with 80.6% of the isolates, before *S*. *cerevisiae* finally became the dominant species in all the samples analyzed after day 16, representing 92.2%, 90.0% and 97.5% of the isolates on days 20, 25 and 34 respectively (Fig 3).

## Discussion

In the present study we evaluated yeast diversity in the vineyard environment of the hot semi-arid climate of the Texas High Plains, and the presence and dynamics of potential enological indigenous yeast populations during the spontaneous fermentation of grapes from their original vineyards.

Yeast diversity in the vineyard environment was found to be higher in soil samples than in leaf or berry samples, and the soil population was differentiable from the two other types of samples (Fig 1). Moreover, it was possible to discriminate the yeast populations based only on the vineyard’s soil. While *A*. *pullulans* and *Cryptococcus* species were recovered from all types of samples, *Naganishia* species, *Solicoccozyma aeria* and *Papiliotrema terrestris* were more specifically associated with soil samples, and only scarcely recovered from the leaf and berry samples (Table 2). Furthermore, *Lecythophora*, *Rhodotorula* and *Cystobasidium* species were exclusively recovered from soil samples. These different species have previously been recovered from the soil of vineyards, pasture and rhizosphere in various independent locations [11, 30, 31]. *A*. *pullulans* was the major species recovered from all berry samples (more than 50% of the isolates identified), where the overall yeast diversity was very low, with only seven different species recovered from all berry samples, and as few as two species identified in sample BGT (Table 1). The other species recovered from the berry samples included *Cryptococcus*, *Filobasidium* and *Naganishia* species. One isolate of *Solicoccozyma aeria*, generally recovered from soil samples, was recovered from one of the berry samples. The low yeast diversity on grapes could be attributed to the isolation method including only a washing of the grape berries. Other studies have shown higher yeast diversity on grapes, but the isolation method included a step of crushing the berries [32–34]. This crushing step selects for potential yeast of interest for enology, such as ascomycetous species *Hanseniaspora*, *Metschnikowia*, *Pichia*, *Saccharomyces*, or many other fermenting yeasts able to survive in grape must. On the other hand, some other yeast species such as *A*. *pullulans* cannot survive in the new conditions after crushing, *i*.*e*. in must and wine [35], and inferences on grape biodiversity are thus biased. Moreover, although *A*. *pullulans* has no real enological impact, it is of significant interest in the vineyard, as it has been shown as a natural biocontrol agent against *Aspergillus carbonarius* contamination, accountable for the sour rot disease, with the ability to reduce Ochratoxine A levels on grapes [36, 37].

Using a recovery method including a washing step instead of a crushing step, we believe that we have a more accurate vision of the biodiversity of the main yeast species present on grapes, leaves and in soils, as some yeast species cannot survive the environmental pressure provided by the release of grape juice. However, we must not rule out having missed some minor species, despite the large number of isolates analyzed. Within the 799 isolates recovered from soils, leaves and berries, one isolate identified as *L*. *thermotolerans* was of potential enological interest. This is, to our knowledge, the first time *L*. *thermotolerans* has been recovered from soil samples.

Spontaneous fermentation experiments for the 2015 vintage were set up as the enology directed counterpart of the vineyard environment biodiversity experiment. As mentioned previously, the must environment selects for yeasts of enological interest. Harvest day analysis of the must (day 0) after berry crushing showed similar results to the biodiversity experiment conducted the previous year on the same vineyards, i.e. a majority of isolates from both batches were identified as *A*. *pullulans* (Fig 3). The population of *A*. *pullulans* then rapidly decreased and disappeared by day 6 after harvest in both batches. Three species of potential enological interest were then recovered, *Hanseniaspora sp*., *L*. *thermotolerans*, and *S*. *cerevisiae*. *Hanseniaspora sp*. and *L*. *thermotolerans* were recovered from both musts, and *S*. *cerevisiae* from the must of batch B. Although never isolated during the sampling of grapes, leaves or soils the previous year, we cannot concluded that *Hanseniaspora sp*. and *S*. *cerevisiae* were absent of the vineyard environment, but could have been missed as low frequency species. On the other hand, we can exclude a potential cellar contamination of our batches with enological yeasts due to the experimental design including sterile equipment and a facility never used before for winemaking experiments. Although *Hanseniaspora* species were never recovered as the dominant species in the must, many studies have shown their presence and importance on the taste and flavor of wine, with the production of volatile metabolites, glycerol or acetate [38–41]. Spontaneous fermentation was observed in both batches (Fig 2). In batch A, the exclusive domination of *L*. *thermotolerans* suggests this species was the unique fermentative yeast. In batch B, alternating dominance between *L*. *thermotolerans* and *S*. *cerevisiae* could suggest co-fermentation by both species. However, fermentation by *L*. *thermotolerans* as the unique fermenting species resulted in an incomplete fermentation in batch A (36.77 g/L of glucose/fructose at the end of fermentation and an alcohol content of 11.6%v/v, for 26.1 Brix at harvest), while potential co-fermentation by both *L*. *thermotolerans* and *S*. *cerevisiae* in batch B resulted in a complete alcoholic fermentation (1.83 g/L of glucose/fructose at the end of fermentation and an alcohol content of 12.9%v/v, for 23.2 Brix at harvest).

*L*. *thermotolerans* (formerly named *Kluyveromyces thermotolerans*) has recently attracted attention due to its ability to acidify the must by producing L-lactic acid [42]. *L*. *thermotolerans* has been shown to improve wine quality while naturally acidifying the wine in co-inoculation or sequential inoculation with *S*. *cerevisiae* [12, 13, 43], and can provide a good alternative to malolactic fermentation in hot climates when combined with *Schizosaccharomyces pombe* [16]. Grape acidity is lower in hot climates and climate changes could increase the difficulties linked to low acidity must and wine, including microbial spoilage and sensory deviations [44, 45]. The Texas High Plains climate is hot and semi-arid, and winemakers work with low acidity musts, as shown in the present study with Tempranillo grapes reaching a pH of 4 at harvest. A complete characterization at the strain level of *L*. *thermotolerans*, *S*. *cerevisiae* and *Hanseniaspora* species isolates recovered in the present study, as well as individual technological screening (mono-inoculation), should provide further knowledge on the enological potential of these indigenous yeast strains. These findings could confirm previous studies on *L*. *thermotolerans*, and could support the utility of *L*. *thermotolerans* as a natural alternative to chemical acidification of musts by addition of tartaric acid. This study could lead to the development of specifically adapted autochthonous starter cultures for winemaking in Texas High Plains, a concept recently proposed for North Apulian wines in Italy [46, 47]. Moreover, the use of a combination of indigenous *S*. *cerevisiae* and *L*. *thermotolerans* as starter cultures could provide a stronger identity to Texas High Plains wines, a necessary step for international recognition of Texas High Plains winemaking industry [48].

One of the challenges in the wine industry is to understand the importance of microbial diversity in local vineyards as a specific component of terroir. Recent studies and reviews have discussed the importance of the wine microbiota and the impact of the different species on final wine sensory characteristics and local specificities [23, 24, 49, 50]. To our knowledge, the present study is the first to focus on the hot semi-arid climate of the emerging Texas High Plains AVA and has shown promising results. Our findings suggest that some specific indigenous yeast species from the Texas High Plains vineyards could be well adapted for the specific chemical composition of juice and must from grapes grown in the area, through the acidification of musts with low acidity by indigenous *L*. *thermotolerans* complemented with indigenous *S*. *cerevisiae* to complete alcoholic fermentation. Although not recovered directly from the surface of the berries, our experimental design of sterile sampling and sanitation protocol gives us confidence that the three species of enological interest recovered during fermentation were not the result of environmental contamination of the must. Instead, we suggest that those species were not recovered on berries due to the limit of detection of our culture method, potentially combined with a possible viable but not cultivable state on berries. As the two parts of the study were made on different years, we also cannot exclude vintage fluctuations of the yeast populations as a potential reason of the discrepancy between vineyard populations and fermentation populations. Development of such starter cultures from indigenous strains could provide better control than spontaneous fermentations while respecting the specific microbial terroir of the area, thus potentially offering a more characteristic identity to the local wines, compared to the use of widely available commercial active dry yeast strains.
